# Evaluation of antiretroviral therapy effect and prognosis between HIV-1 recent and long-term infection based on a rapid recent infection testing algorithm

**DOI:** 10.3389/fmicb.2022.1004960

**Published:** 2022-11-22

**Authors:** Jianhui Zhao, Hongjie Chen, Zhengwei Wan, Tao Yu, Quanxun Liu, Jingwei Shui, Haiying Wang, Jie Peng, Shixing Tang

**Affiliations:** ^1^Department of Epidemiology, School of Public Health, Southern Medical University, Guangzhou, China; ^2^Department of Infectious Diseases, Nanfang Hospital, Southern Medical University, Guangzhou, Guangdong Province, China; ^3^Department of Health Management and Institute of Health Management, Sichuan Provincial People's Hospital, University of Electronic Science and Technology of China, Chengdu, China

**Keywords:** HIV-1, recent infection, long-term infection, point-of-care testing, recent infection testing algorithms, clinical prognosis

## Abstract

Early diagnosis of HIV-1 infection and immediate initiation of combination antiretroviral therapy (cART) are important for achieving better virological suppression and quicker immune reconstitution. However, no serological HIV-1 recency testing assay has been approved for clinical use, and the real-world clinical outcomes remain to be explored for the subjects with HIV-1 recent infection (RI) or long-term infection (LI) when antiretroviral therapy is initiated. In this study, a HIV-1 rapid recent-infection testing strip (RRITS) was developed and incorporated into the recent infection testing algorithms (RITAs) to distinguish HIV-1 RI and LI and to assess their clinical outcomes including virological response, the recovery of CD4^+^ T-cell count and CD4/CD8 ratio and the probability of survival. We found that the concordance between our RRITS and the commercially available LAg-Avidity EIA was 97.13% and 90.63% when detecting the longitudinal and cross-sectional HIV-1 positive samples, respectively. Among the 200 HIV-1 patients analyzed, 22.5% (45/200) of them were RI patients and 77.5% (155/200) were chronically infected and 30% (60/200) of them were AIDS patients. After cART, 4.1% (5/155) of the LI patients showed virological rebound, but none in the RI group. The proportion of CD4^+^ T-cell count >500 cells/mm^3^ was significantly higher in RI patients than in LI after 2 years of cART with a hazard ratio (HR) of 2.6 (95% CI: 1.9, 3.6, *p* < 0.0001) while the probability of CD4/CD8 = 1 was higher in RI than in LI group with a HR of 3.6 (95% CI: 2.2, 5.7, *p* < 0.0001). Furthermore, the immunological recovery speed was 16 cells/mm^3^/month for CD4^+^ T-cell and 0.043/month for the ratio of CD4/CD8 in the RI group, and was bigger in the RI group than in the LI patients (*p* < 0.05) during the 1st year of cART. The survival probability for LI patients was significantly lower than that for RI patients (*p* < 0.001). Our results indicated that RRITS combined with RITAs could successfully distinguish HIV-1 RI and LI patients whose clinical outcomes were significantly different after cART. The rapid HIV-1 recency test provides a feasible assay for diagnosing HIV-1 recent infection and a useful tool for predicting the outcomes of HIV-1 patients.

## Introduction

Successful control of HIV-1 epidemics relies on early diagnosis of HIV-1 infection, immediate initiation of combination antiretroviral therapy (cART) and effective suppression of HIV-1 replication during cART, which are the target goals proposed by The Joint United Nations Program on HIV and AIDS (UNAIDS) (2020). It has been well documented that initiation of cART in early HIV-1 infection could result in rapid immunological recovery and better clinical efficacy (Fiebig et al., 2003; Le et al., 2013). In addition, several studies have proved the beneficial effects of starting cART during primary or recent HIV-1 infection on disease progression, such as reducing viremia (Gianella et al., 2011), limiting the size of virus latent pool (Jain et al., 2013), and preserving immune system (Le et al., 2013; Davy-Mendez et al., 2018). Therefore, differentiating HIV-1 recent infection (RI) and long-term infection (LI) may help precisely manage antiretroviral therapy and monitor cART efficacy.

At present, longitudinal sampling and testing of uninfected individuals are the “gold-standard” approach to identify recent HIV-1 infections. However, it is difficult, time-consuming, expensive and technically infeasible (Gable and Lagakos 2008 ). Several types of serological assays and new biomarkers have recently been developed to differentiate RI and LI of HIV-1, such as less-sensitive enzyme immunoassay (LS-EIA; Rawal et al., 2003), the BED capture enzyme immunoassay (BED-CEIA; Dobbs et al., 2004) and limiting antigen avidity enzyme immunoassay (LAg-Avidity EIA; Duong et al., 2012). Among these assays, BED-CEIA and LAg-Avidity EIA were commercially available and commonly used. Furthermore, HIV-1 recency testing assay has been incorporated into the recent infection testing algorithms (RITAs) and recommended by UNAIDS to differentiate HIV-1 patients with RI and LI (Organization W.H, 2011). However, most serological assays, including LAg-Avidity EIA and BED-CEIA, still depend on the laboratory facility and professional operator, which in turn limit their implementation in point-of-care testing (POCT) and resource-limiting settings.

We have previously identified a 57-mer peptide (gp41-p57) located at the loop region of HIV-1 gp41 protein that could differentiate HIV-1 RI and LI (Li et al., 2016). Our results also confirmed that the recombinant protein MP4 containing HIV-1 gp41 immunodominant epitopes (IDEs) of the major HIV-1 genotype CRF01_AE, CRF07_BC/CRF08_BC and subtype B in China could inhibit the binding of gp41 peptide with anti-HIV antibody of low avidity, and was suitable for distinguishing HIV-1 RI and LI (Cai et al., 2019). The MP4-based enzyme-linked immunosorbent assay showed comparable performance with LAg-Avidity EIA (Cai et al., 2019). In the current study, we reported a HIV-1 rapid recent-infection testing strip (RRITS) assay for distinguishing HIV-1 RI and LI based on the gp41 recombinant antigen MP4 and BE23 and evaluated its clinical utility alone or integrated into the RITAs recommended by UNAIDS to differentiate RI and LI patients. By implementing the rapid HIV-1 recency testing assay in a general hospital in Guangzhou, China, we identified substantial chronically HIV-infected patients whose clinical outcomes after cART were quite different from those with recent HIV-1 infection. Our results confirmed the clinical utility of rapid HIV-1 recency testing assay in precisely identifying and managing HIV-1 patients.

## Materials and methods

### Specimens for the evaluation of HIV-1 RRITS

The specimens used for the evaluation included three panels (Supplementary Table S1). The optimization of our HIV-1 recency testing RRITS was performed using specimens of Panel 1 that contains 118 samples including 98 anti-HIV positive (11 CRF01_AE, 31 CRF07_BC, 14 CRF08_BC, 3 Subtype B, 39 Unknown) and 20 anti-HIV negative healthy volunteer samples obtained from Beijing Xinchuang Bioengineering Co., Ltd. (Beijing, China) and detected by anti-HIV enzyme-linked immunosorbent assay (ELISA) of WANTAI BioPharm (Beijing, China). HIV-1 positive samples were further classified into HIV-1 RI and LI groups by using the commercial BED-CEIA (Sedia Biosciences, Portland, OR, United States) and LAg-Avidity EIA (Kinghawk Pharmaceutical Co., Ltd., Beijing, China) kits. Panel 2 included 36 archived de-linked serum samples from 9 patients undergoing acute HIV-1 seroconversion. They were prospectively collected and have been previously described (Cai et al., 2019), and were used to calculate the mean duration of recent infection (MDRI) of our RRITS assay. Each patient provided 4 follow-up samples from the last date with anti-HIV negative result (day 0) up to 602 days after HIV-1 seroconversion. Panel 3 included 110 specimens (40 CRF01_AE, 40 CRF_07BC, 7 Subtype B and 23 CRF55_01B) from the Guangzhou Center for Disease Control and Prevention, 200 from Nanfang Hospital of Guangzhou and 85 from the Guangzhou Eighth People’s Hospital. These samples were detected by the recency test of HIV-1 LAg-Avidity EIA Kit and used to further validate the performance of our HIV-1 RRITS.

### Patients

To evaluate the impact of HIV-1 recency testing on disease progress and outcome, 200 HIV-1 patients from Nanfang Hospital, a general teaching hospital of Southern Medical University in Guangzhou, China. In our study, 200 HIV-1 patients were selected from 536 HIV-1 infected patients through simple random sampling in Nanfang Hospital during September 2018 and November 2019. Baseline characteristics, including gender, age, mode of infection, marital status and cART regimen were collected from clinical records. The CD4^+^ and CD8^+^ T-cell counts, plasma HIV-1 RNA level and other laboratory data were collected. Samples of whole blood and plasma were collected and frozen at −80°C. Virological suppression was defined as a plasma HIV-1 RNA load of <50 copies/mL (CMA and CCDC, 2022). Virological rebound was defined as HIV-1 RNA level ≥ 200 copies/mL after virological suppression (CMA and CCDC, 2022). AIDS patients were defined as HIV-1 patients with CD4^+^ T-cell counts <200 cells/mm^3^ and/or pneumocystis yersini pneumonia (CMA and CCDC, 2022). The clinical study was approved by the Nanfang Hospital Ethics Committee (NFEC-2021-448), and all patients provided informed consent.

### Recent infection testing algorithms

**Figure 1:**
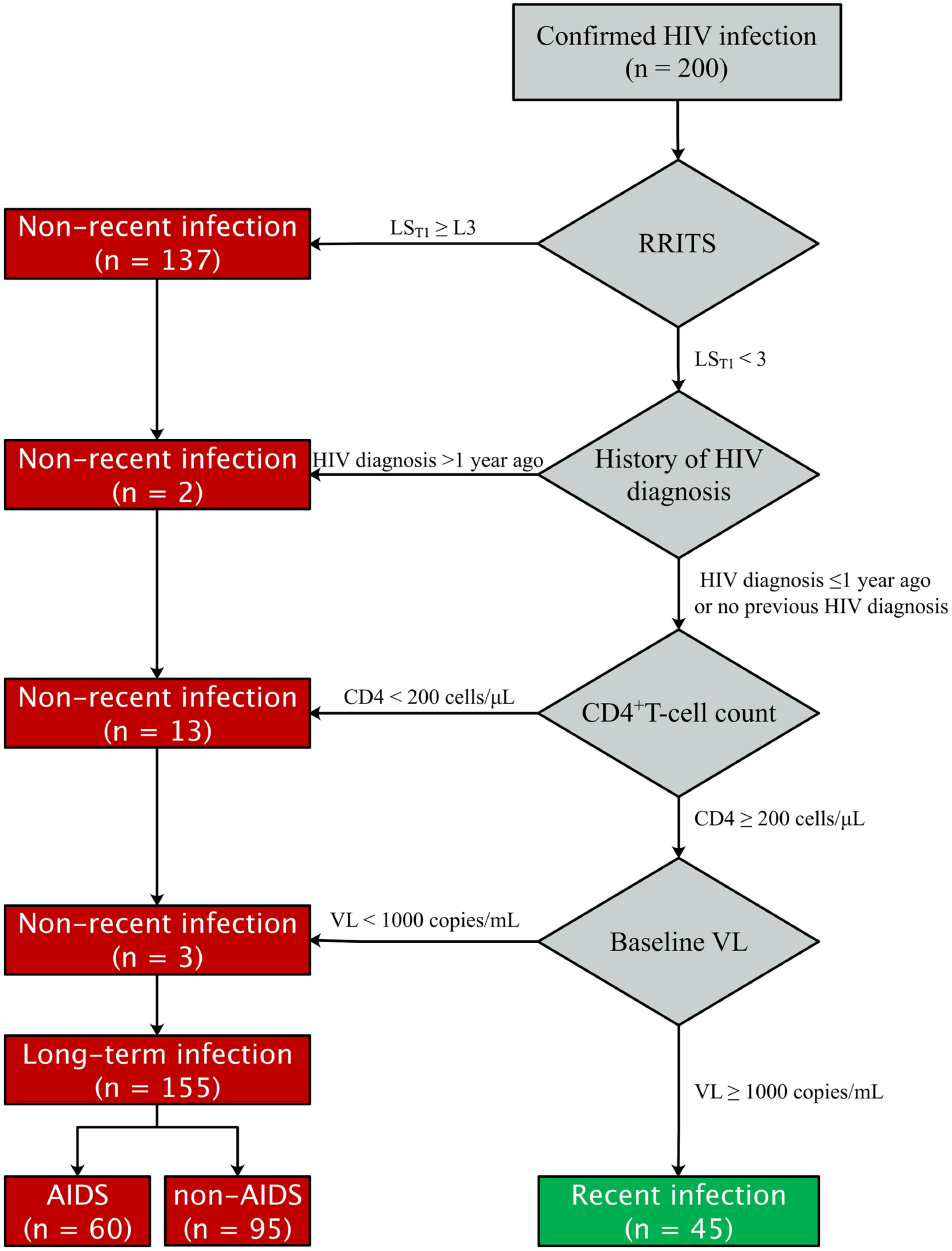
Flow chat of HIV-1 recent infection testing algorithms (RITAs) to classify recent and long-term HIV-1 infection. RRITS, rapid recent-infection testing strip; LS_T1_, the T_1_ line score of RRITS for detection of long-term HIV-1 infection; HIV, human immunodeficiency virus; AIDS, acquired immunodeficiency syndrome; VL, viral load.

The flow chart of RITAs was adapted from the file of UNAIDS/WHO Working Group (Organization W.H, 2011) and shown in Figure 1. The criteria for HIV-1 RI patients included: (1) HIV-1 RRITS line score T_1_ < L3; Of note, T_1_ line was specifically designed to distinguish HIV-1 RI and LI according to its density or score since the coating antigen in T1 line can specifically bind mature anti-gp41 antibody with high avidity, which is usually found in HIV-1 long-term infection, but not in recent infection. (2) HIV-1 diagnosis ≤ 1 year (Rice et al., 2020; de Wit et al., 2021; Teixeira et al., 2021); (3) baseline CD4^+^ T-cell counts ≥ 200 cells/μl (Chauhan et al., 2020; Karatzas-Delgado et al., 2020; Zhu et al., 2020; Ang et al., 2021; Teixeira et al., 2021); (4) baseline HIV-1 RNA level ≥ 1,000 copies/ml (Rice et al., 2020; Zhu et al., 2020; de Wit et al., 2021; Rwibasira et al., 2021; Voetsch et al., 2021). Otherwise, the HIV-1 patients were considered as long-term infection (LI) of HIV-1. The development, evaluation and testing of RRITS method were presented in the Supplementary methods.

### Prediction model of mortality of cART-treated HIV-1 patients

The parameters of HIV-1 mortality risk model (Hou et al., 2019) was obtained from the nomogram with GetData Graph Digitzer (Version 2.5). The prognostic index for each patient (i) was calculated using the following formula:


$$\begin{array}{c} {\mathrm{PI}}_{i}=-0.005580907\times \mathrm{CD}4\;\mathrm{cell count}-0.005368102\times \mathrm{hemoglobin}\\ +1.019669556\;\left[\mathrm{if}\;\mathrm{HIV}\;{\mathrm{viral load}}_{i}\;\mathrm{is within}\;\;200-1000\right]\\ +2.608969326\left[\mathrm{if}\;\mathrm{HIV}\;{\mathrm{viral load}}_{i}\geq 1000\right] \end{array}$$

The predicted survival probability at *t* year (*t* = 1, 2, and 3) after cART initiation for each patient (i) was determined using the following formula:


$$\hat{S_{i}}\;\left(1\right)=0.980222074^{\exp\left({\mathrm{PI}}_{i}\right)}$$

$$\hat{S_{i}}\;\left(2\right)=0.972736744^{\exp\left({\mathrm{PI}}_{i}\right)}$$

$$\hat{S_{i}}\;\left(3\right)=0.964896148^{\exp\left({\mathrm{PI}}_{i}\right)}$$

The risk score for each patient (i) was calculated using the following formula:


$${\mathrm{Risk score}}_{i}=108.3333333+19.90914787\times {\mathrm{PI}}_{i}$$

The adjusted prognostic index by age and gender for each patient (i) was calculated using the following formula (Wang et al., 2020):


$$\begin{array}{c} \begin{array}{l} {\mathrm{PIadjust}}_{i}={\mathrm{PI}}_{i}+0.2382292\;\left[\mathrm{if}\;{\mathrm{age}}_{i}\;\mathrm{is within}\;\;40-60\right]\\ \;+0.5866749\left[\mathrm{if}\;{\mathrm{age}}_{i}\geq 60\right] \end{array}\\ -0.3566749\left[{\mathrm{if gender}}_{i}=\mathrm{female}\right] \end{array}$$

### Statistical analysis

The data were analyzed with R software version 3.6.2 and STATA/SE 15.0 (STATA Corp, College Station, TX) and graphed with GraphPad Prism 8.0 (GraphPad Software, California, United States). The Chi-square Test/ANOVA analysis and the independent-sample *T*-Test or Mann–Whitney/Kruskal-Wallis H Test were used for qualitative and quantitative variables, respectively. Polynomial regression was used to estimate the mean duration of recent infection (MDRI) of the RRITS, and Spearman correlation was used to analyze the correlation between line score T_1_ and ODn. The recovery speed of CD4^+^ T-cell count and CD4/CD8 ratio was compared between the RI and LI groups by using the Log-rank test, and the hazard ratio (HR) was calculated. The dynamics of CD4^+^ T-cell count and CD4/CD8 ratio for the RI vs. LI groups during cART were compared using generalized estimating equations (GEE). CD4^+^ T-cell count and CD4/CD8 ratio and 95% pointwise confidence bands were obtained from nonlinear GEE. All tests were two-sided and *p* < 0.05 was set as the significant level. Propensity score matching (PSM) was used to conduct a 1:1 case–control study by controlling age, baseline CD4^+^ T-cell count, baseline HIV-1 RNA level, gender, mode of infection, marital status, cART regimen and drug therapy adherence.

## Results

### Development and evaluation of HIV-1 RRITS

**Figure 2:**
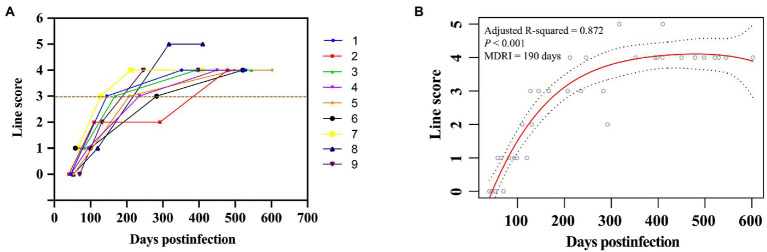
Discrimination of HIV-1 recent and long-term infection using rapid recent-infection testing strip in longitudinal cohort samples. **(A)** A total of 36 longitudinal seroconversion samples collected from 9 HIV-1-infected individuals were labeled with different colors and detected by rapid recent-infection testing strip (RRITS) at different time points. Y axis shows T_1_ line score of RRITS, which is determined according to the density of T_1_ line. The brown dash line represents the cut-off value of T_1_ line of RRITS. **(B)** The relationship between the time post-infection and the line score of T_1_ through polynomial regression analysis. The polynomial regression values and their 95% confidence intervals are presented in red solid line and blue curves, respectively. MDRI, mean duration of recent infection.

We first determined the coating ratio of HIV-1 gp41 recombinant antigen BE23 and MP4, which was 1:3 (Supplementary Figure S1), and the optimal cut-off value of RRITS T1 line as L3 when distinguishing HIV-1 RI and LI (Supplementary Figure S2). Under these conditions, the AUC of the receiver operator characteristic curve was 0.946 (Supplementary Figure S2) and the sensitivity and specificity of RRITS were 98.98% (97/98) and 100.00% (20/20), respectively when compared with anti-HIV ELISA (Supplementary Table S2). Among the 36 seroconversion samples determined to be HIV-1 RI or LI by both Maxim and KingHawk LAg-Avidity EIA, 35 (97.22%) were correctly recognized by the RRITS with a Kappa value of 0.940 (Figure 2A, Table 1). A good correlation between the time of post-infection and the line score of T_1_ was obtained by polynomial regression analysis with an *R*^2^ value of 0.872 (Figure 2B). The estimated MDRI of RRITS was 190 days and the false-recent rate (FRR) was 5.88% (2/34). Furthermore, we compared the performance of RRITS and LAg-Avidity EIA assays in 395 cross-sectional specimens. The Kappa value and concordance rate were 0.806% and 90.63%, respectively (Table 1). As shown in Supplementary Figure S3, a good correlation between the line score of T_1_ for our RRITS and ODn of LAg-Avidity EIA was observed. The correlation coefficient was 0.843 (*p* < 0.001). Furthermore, our results indicated that HIV-1 RRITS showed a comparable detection capapbility for the predominant HIV-1 genotypes ro subtypes in China (Supplementary Table S3).

**Table 1:** Comparison of rapid recent-infection testing strip (RRITS) and LAg-Avidity EIA to distinguish HIV-1 recent and long-term infection.

Groups	Methods		LAg-Avidity EIA		Total	Kappa value	Concordance (%)	Predictive Value (%)	
			RI	LI				RI	LI
Longitudinal samples	RRITS	RI	13	1	14	0.940	97.13	92.86	100.00
		LI	0	22	22				
		Total	13	23	36				
Cross-sectional samples^a^		RI	142	11	153	0.806	90.63	92.81	89.26
		LI	26	216	242				
		Total	168	227	395				

a 395 cross-sectional serum samples from multiple-center studies, including Guangzhou Center for Disease Control and Prevention, The Eighth People’s Hospital of Guangzhou and Nanfang Hospital of Guangdong. RI, recent infection; LI, long-term infection.

### Comparison of RITAs and rapid HIV-1 recency testing alone to differentiate HIV-1 recent infection and long-term infection

31.5% (63/200) and 68.5% (137/200) HIV-1 patients were identified as RI and LI, respectively based on RRITS alone while 26.5% (53/200) and 73.5% (147/200) patients were RI and LI, respectively based on the results of RITAs and LAg-Avidity EIA (Supplementary Figure S4). Although the difference of RI and LI identified by RRITS alone or RITAs was statistically significant (*p* < 0.01), the results of the two methods were correlated well (Kappa = 0.774, Supplementary Table S4). Of note, further analysis showed no significant difference in virological response, immune reconstitution and prognostic risk score between HIV-1 RI and LI groups differentiated by RITAs using RRITS or LAg-Avidity EIA and RRITS alone after cART (*p* > 0.1, Table 2).

**Table 2:** Analysis of virological response, immune reconstitution and prognostic risk score in 200 cART-treated HIV-1 patients.

Variable	Differentiation strategies			Value of *p*^c^	Value of *p*^d^	Value of *p*^e^
	RRITS	RITAs1^a^	RITAs2^b^			
*HIV-1 patients of recent infection*						
Attainment of virological suppression at 6 months after cART	54 (91.5%)	40 (95.2%)	46 (93.9%)	0.744	0.924	>0.999
Attainment of virological suppression at 12 months after cART	56 (96.6%)	40 (97.6%)	46 (95.8%)	>0.999	>0.999	>0.999
Virological rebound at 12 months after cART	0 (0.0%)	0 (0.0%)	1 (2.2%)	-	0.465	>0.999
CD4^+^ T-cell recovery speed (cells/mm^3^/mo) at 1st year of cART	15	16	16	0.384	0.175	0.441
CD4/CD8 recovery speed per month at 1st year of cART	0.036	0.042	0.040	0.152	0.175	0.363
CD4^+^ T-cell recovery speed (cells/mm^3^/mo) at 2nd year of cART	7	8	8	0.257	0.318	0.416
CD4/CD8 recovery speed per month at 2nd year of cART	0.012	0.015	0.015	0.257	0.271	0.465
Prognostic risk score, P_50_ (P_25_, P_75_)	108 (98, 118)	100 (93, 111)	101 (95, 111)	0.722	0.787	>0.999
*HIV-1 patients of long-term infection*						
Attainment of virological suppression at 6 months after cART	109 (92.4%)	123 (91.1%)	117 (91.4%)	0.717	0.782	0.933
Attainment of virological suppression at 12 months after cART	107 (93.9%)	123 (93.9%)	117 (94.4%)	0.932	>0.999	0.935
Virological rebound at 12 months after cART	5 (4.6%)	5 (4.1%)	4 (3.5%)	>0.999	0.893	>0.999
CD4^+^ T-cell recovery speed (cells/mm^3^/mo) at 1st year of cART	10	11	10	0.406	0.395	0.492
CD4/CD8 recovery speed per month at 1st year of cART	0.024	0.024	0.024	0.496	0.423	0.446
CD4^+^ T-cell recovery speed (cells/mm^3^/mo) at 2nd year of cART	4	4	4	0.452	0.489	0.443
CD4/CD8 recovery speed per month at 2nd year of cART	0.010	0.009	0.010	0.448	0.395	0.478
Prognostic risk score, P_50_ (P_25_, P_75_)	119 (100, 128)	116 (102, 127)	121 (102, 128)	>0.999	>0.999	>0.999

a RITAs1 was based on HIV-1 rapid recent-infection testing strip (RRITS).

b RITAs2 was based on LAg-Avidity EIA.

c Value of *p* was calculated for RRITS vs. RITAs1.

d Value of *p* was calculated for RRITS vs. RITAs2.

e Value of *p* was calculated for RITAs1 vs. RITAs2.

### Differentiation of recent and long-term HIV-1 infection

UNAIDS recommends the combination of RITAs with HIV-1 serological recency testing and routine clinical tests to more accurately distinguish RI and LI (WHO, 2011; accessed January 23, 2022). Thus, we integrated the history of HIV-1 diagnosis, baseline CD4^+^ T-cell count and HIV-1 RNA level and our RRITS results to determine the infection status of HIV-1 patients (Figure 1). Among the 200 patients analyzed, 45 (22.5%) and 155 (77.5%) patients were identified as RI and LI, respectively while 60 (30%) were AIDS patients. Clinical and demographical characteristics between RI and LI groups were shown in Table 3, and no significant difference was observed between the RI and LI patients in the mode of infection, marital status, cART regimen, cART adherence and baseline HIV-1 RNA level. Only the proportion of female patients was higher in long-term infections than males (95.2% vs. 75.4%).

**Table 3:** Clinical and demographical characteristics of the study cohort.

Variables	RI (*n* = 45)	LI (*n* = 155)	Total (*n* = 200)	Value of *p*^a^
Age (year), median (IQR)	25.0 (22.0, 34.0)	31.0 (25.0, 41.0)	30.0 (24.0, 39.8)	<0.001
Gender, no. (%)				0.075
Female	1 (2.2)	20 (12.9)	21 (10.5)	
Male	44 (97.8)	135 (87.1)	179 (89.5)	
Mode of infection, no. (%)				0.183
Heterosexual contact	4 (8.9)	31 (20.0)	35 (17.5)	
Homosexual contact	41 (91.1)	122 (78.7)	163 (81.5)	
Unknow	0 (0.0)	2 (1.3)	2 (1.0)	
Marital status, no. (%)				0.223
Married or cohabiting	8 (17.8)	46 (29.7)	54 (27.0)	
Divorced or separated or widowed	5 (11.1)	20 (12.9)	25 (12.5)	
Unmarried	32 (71.1)	89 (57.4)	121 (60.5)	
cART regimen, no. (%)				0.897
TDF + 3TC + EFV	43 (95.6)	144 (92.9)	187 (93.5)	
TDF + 3TC + LPV/r	0 (0.0)	1 (0.7)	1 (0.5)	
TDF + 3TC + DTG	1 (2.2)	7 (4.5)	8 (4.0)	
E + C + T + F	1 (2.2)	3 (1.9)	4 (2.0)	
cART adherence, no. (%)				0.676
Optimal	35 (77.8)	111 (71.6)	146 (73.0)	
Sub-optimal	10 (22.2)	41 (26.5)	51 (25.5)	
Unknow	0 (0.0)	3 (1.9)	3 (1.5)	
Baseline CD4^+^ T-cell count (cells/mm^3^), median (IQR)	391.0 (294.5, 462.0)	155.5 (244.0, 377.5)	284.5 (182.8, 411.3)	<0.001
Baseline HIV-1 RNA level (log_10_ copies/ml), median (IQR)	4.20 (3.77, 4.82)	3.69 (4.20, 4.77)	4.20 (3.73, 4.80)	0.522

RI, recent infection; LI, long-term infection; TDF, tenofovir disoproxil fumarate; 3TC, lamivudine; EFV, efavirenz; LPV/r, lopinavir/ritonavir; E, elvitegravir; C, cobicistat; T, tenofovir; F, emtricitabine; cART, combination antiretroviral therapy; IQR, interquartile range.

a Comparison between groups before cART: Mann–Whitney test, or *χ*^2^ test or Fisher exact test.

### Difference in virological response and immune-reconstitution between the patients with recent and long-term HIV-1 infection after cART

We then compared RI and LI patients in virological response and immune reconstitution during cART. The results indicated that 95.2% of RI and 91.1% of LI patients reached virological suppression after 6 months of cART while 97.6% of RI and 93.9% of LI patients were virologically suppressed after 12 months of cART (Table 4). Interestingly, 4.1% of LI patients showed virological rebound at 12 months post cART, but no RI patients relapsed although the difference was not statistically significant (*p* = 0.336, Table 4). We further divided LI patients into LI with AIDS (LI AIDS) and without AIDS (LI non-AIDS) according to the Chinese Guidelines for Diagnosis and Treatment of HIV/AIDS (2021) (CMA and CCDC, 2022), and found a lower virological suppression rate in the LI AIDS, i.e., 85.7% at 6 months of cART and 90.6% at 12 months of cART, than LI non-AIDS patients (Table 5). Furthermore, a slightly higher frequency of virological rebound was observed in LI AIDS (6.4%) and LI non-AIDS (2.7%) patients during 12 months of cART when compared to the RI group (Table 5).

**Table 4:** Virological suppression and rebound in 200 cART-treated HIV-1 patients of recent or long-term infection.

Variable	No. (%)			Value of *p*^a^
	Total	RI	LI	
Attainment of virological suppression^b^ at 6 months after cART	163 (92.1)	40 (95.2)	123 (91.1)	0.524
Attainment of virological suppression at 12 months after cART	163 (94.8)	40 (97.6)	123 (93.9)	0.688
Virological rebound^c^ at 12 months after cART	5 (3.1)	0 (0.0)	5 (4.1)	0.336

cART, combination antiretroviral therapy; RI, recent infection; LI, long-term infection.

a Values of *p* were calculated by chi-square test.

b Virological suppression was defined as HIV-1 viral load < 50 copies/ml.

c Virological rebound was defined as HIV-1 viral load increased from < 50 copies/ml at 6 months after cART to ≥200 copies/ml at 12 months after cART.

**Table 5:** Virological suppression and rebound in 200 cART-treated HIV-1 patients of recent or long-term infection with or without AIDS.

Variable	No. (%)			Value of *p*^a^	Value of *p*^b^	Value of *p*^c^
	RI	LI (non-AIDS)	LI (AIDS)			
Attainment of virological suppression at 6 months after cART	40 (95.2)	75 (94.9)	48 (85.7)	0.181	>0.999	0.122
Attainment of virological suppression at 12 months after cART	40 (97.6)	75 (96.2)	48 (90.6)	0.227	>0.999	0.268
Virological rebound at 12 months after cART	0 (0.0)	2 (2.7)	3 (6.4)	0.248	0.544	0.375

a Value of *p* was calculated for RI vs LI (AIDS).

b Value of *p* was calculated for RI vs LI (non-AIDS).

c Value of *p* was calculated for LI (AIDS) vs LI (non-AIDS).

**Figure 3:**
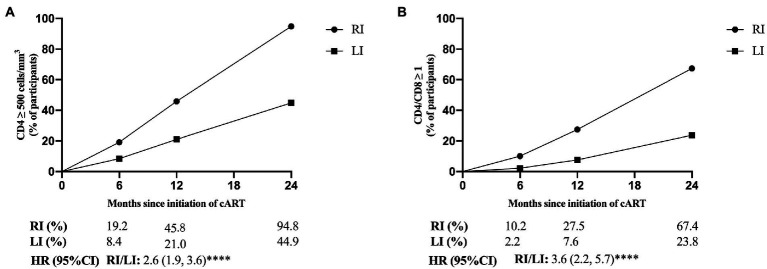
The proportion of CD4^+^ T-cell count ≥ 500 cells/mm^3^ and CD4/CD8 ratio ≥ 1 for HIV-1 patients with recent and long-term HIV-1 infections after cART. CD4^+^ and CD8^+^ T-cell counts **(A)** and the ratio of CD4/CD8 **(B)** were detected in 200 cART-treated patients at different time points. RI, recent infection; LI, long-term infection; HR, hazard ratio; *, *p* < 0.05; **, *p* < 0.01; ***, *p* < 0.001; ****, *p* < 0.0001; ns, *p* > 0.05.

**Figure 4:**
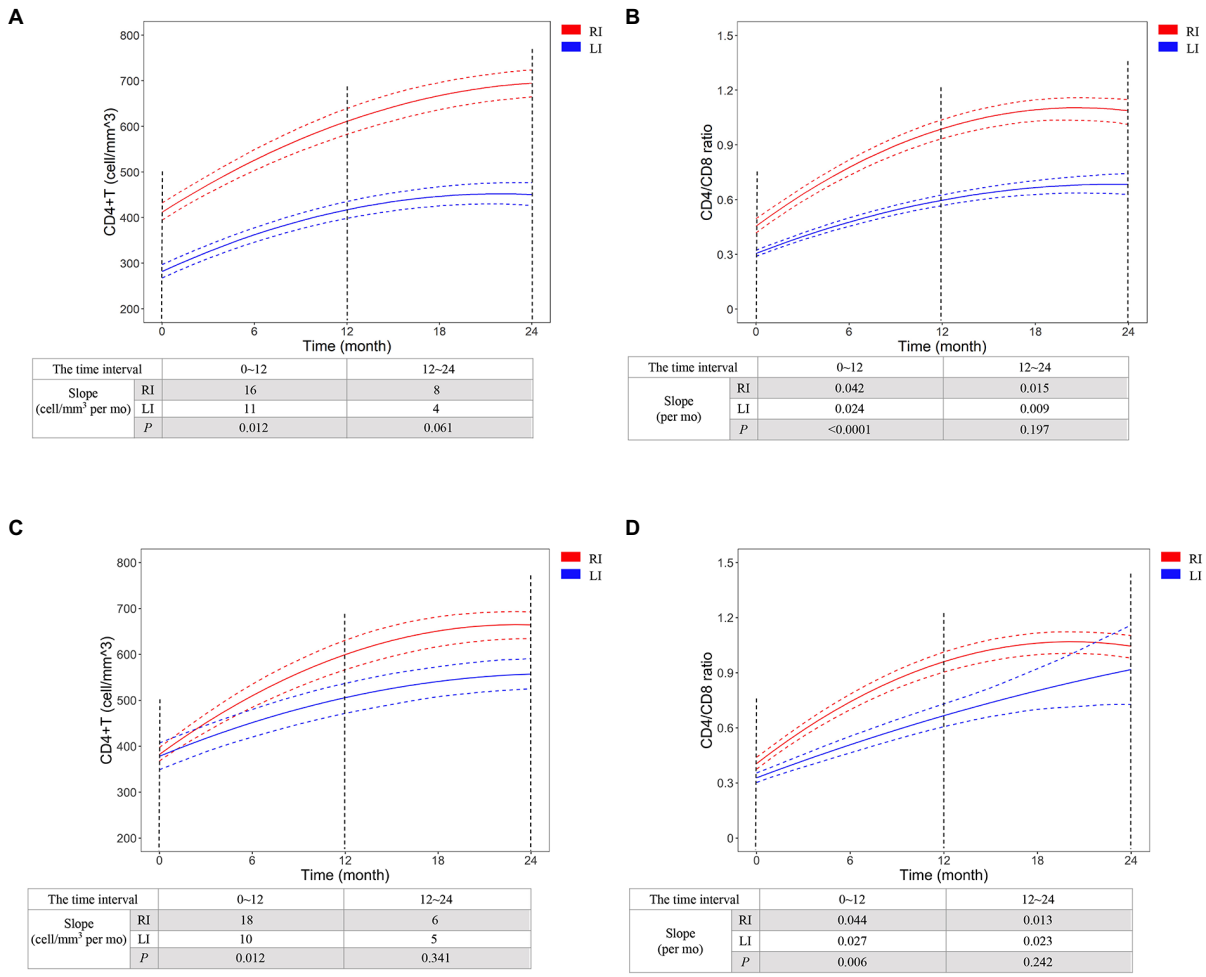
The recovery dynamics of CD4^+^ T-cell count **(A,C)** and CD4/CD8 ratio **(B,D)** after initiation of cART for HIV-1 recent and long-term infection patients. CD4^+^ T-cell count and CD4/CD8 ratio were detected in all 200 cART-treated patients **(A,B)** or the patients of 1:1 case–control analysis using propensity score matching method. The dynamics was predicted by Generalized Estimating Equations (GEE) and compared between HIV-1 recent and long-term infection patients. The red and blue dash lines represent 95% pointwise confidence bands. RI, recent infection; LI, long-term infection. RI, recent infection; LI, long-term infection.

We selected CD4^+^ T-cell count ≥500 cells/mm^3^ and CD4/CD8 ratio ≥ 1 to represent the primary endpoints of immunological reconstruction (Le et al., 2013), and found that 45.2% patients did not meet the criteria of primary CD4^+^ T-cell recovery after 2 years of cART, but 95.7% of them were LI patients (Figure 3A). The probability of CD4^+^ T-cell count recovered to 500 cells/mm^3^ was 2.6 times higher in RI patients than LI (95% CI: 1.9, 3.6, *p* < 0.0001, Figure 3A). Meanwhile, 67.4% of RI and 23.8% of LI patients met the criteria for CD4/CD8 ratio recovery after 2 years of cART (Figure 3B). The probability of CD4/CD8 ratio ≥ 1 was 3.6 times higher in RI patients than LI patients (95% CI: 2.2, 5.7, *p* < 0.0001, Figure 3B). In addition, we found that the recovery speed of CD4^+^ T-cell count was faster in RI patients (16 cells/mm^3^ per month) than LI patients (11 cells/mm^3^ per month, *p* = 0.012, Figure 4A) while a similar trend was observed for the recovery speed of CD4/CD8 ratio, i.e., 0.042 per month for RI patients and 0.024 per month for LI patients (*p* < 0.0001, Figure 4B). Although the recovery speed of the immune reconstruction of RI patients was slightly quicker than LI patients during 12–24 months of cART, the difference was not statistically significant (*p* = 0.061 for CD4^+^ T-cell count; *p* = 0.197 for CD4/CD8 ratio). Furthermore, the CD4^+^ T-cell count recovery speed of RI, LI non-AIDS and LI AIDS patients were 16, 11 and 11 cells/mm^3^ per month, respectively (Supplementary Figure S5A) whereas the corresponding CD4/CD8 ratio recovery speed was 0.042, 0.028 and 0.018 per month, respectively (Supplementary Figure S5B). The recovery speed of CD4^+^ T-cell count and CD4/CD8 ratio at the first 6 months, 6–12 and 12–24 months after cART was analyzed separately and was fastest in the first 6 months of cART for both RI and LI patients (Supplementary Figure S6).

To avoid potential variation, we conducted a 1:1 case–control study based on PSM analysis to adjust the factors of age, baseline CD4^+^ T-cell count, baseline HIV-1 RNA level, gender, mode of infection, marital status, cART regimen and cART adherence (Supplementary Table S5). GEE analysis confirmed the difference of CD4^+^ T-cell count recovery speed between RI and LI patients (18 vs. 10 cells/mm^3^ per month, *p* = 0.012) and CD4/CD8 ratio recovery speed (0.044 vs. 0.027 per month, *p* = 0.006) during 12 months of cART (Figures 4C,D). These results indicated better immunological reconstitution in RI patients during 1st year of cART.

### Prognosis of cART-treated patients with recent or long-term HIV-1 infection

**Figure 5:**
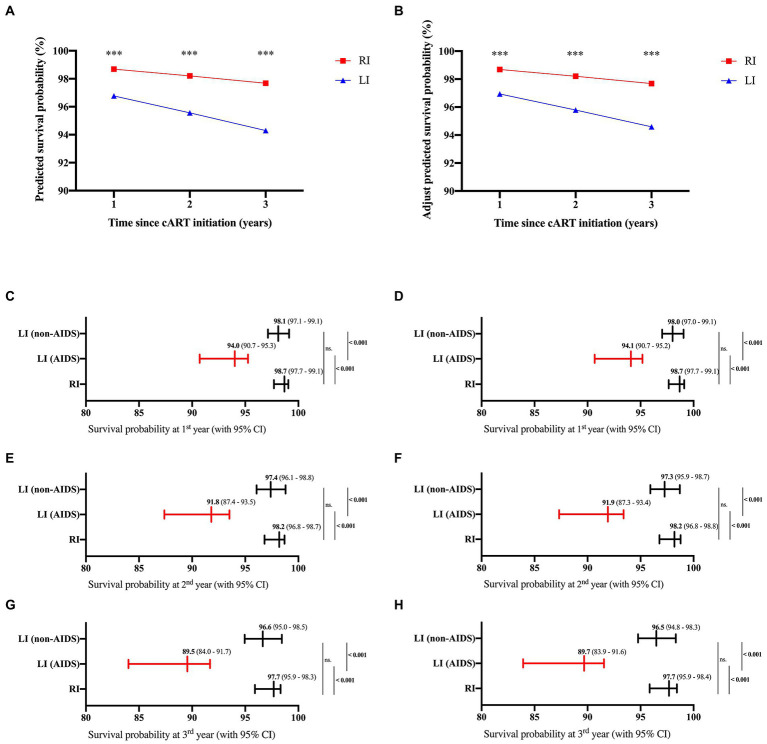
Predicted survival probabilities of cART-treated HIV-1 patients. The survival probabilities were predicted based on Wenzhou model (left) and adjusted for age and gender (right) according to the previous studies (Hou et al., 2019; Wang et al., 2020) for HIV-1 patients of recent and long-term infection at 1st, 2nd and 3rd year after cART **(A,B)**, or for HIV-1 patients of recent and long-term infection with or without AIDS at 1st **(C,D)**, 2nd **(E,F)**, and 3rd **(G,H)** year after cART.

**Figure 6:**
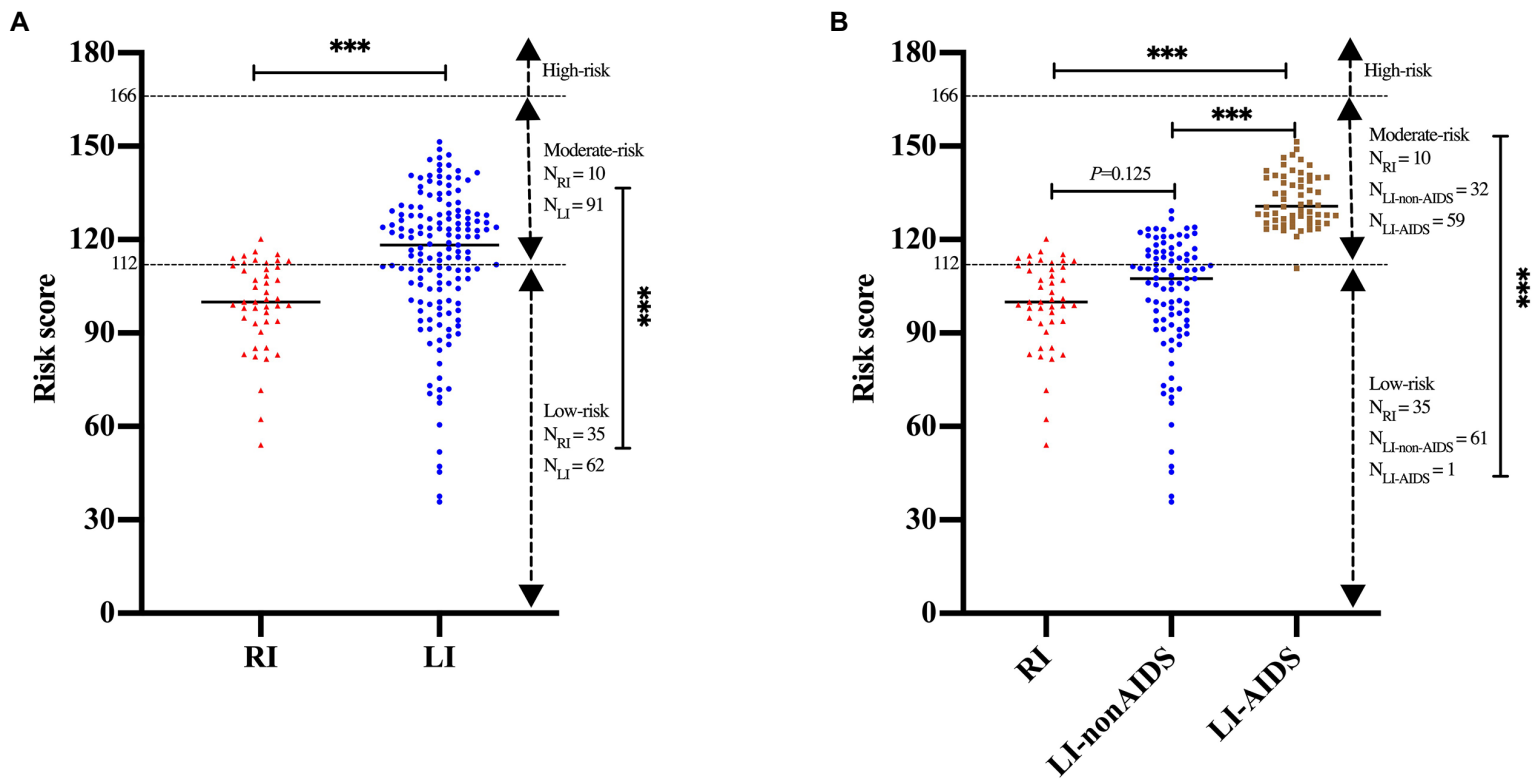
Prognostic risk scores of recent and long-term HIV-1 infection patients. The prognostic scores were calculated to predict mortality using Wenzhou model and were categorized into three risk groups: low (<112), moderate (112∼) and high (≥166) according to the previous studyies (Hou et al., 2019; Wang et al., 2020). **(A)** The prognostic risk scores for HIV-1 patients of recent and long-term infection. **(B)** The prognostic risk scores for HIV-1 patients of recent and long-term infection with or without AIDS. *, *p* < 0.05; **, *p* < 0.01; ***, *p* < 0.001.

No mortality event was observed in our study due to the relatively short follow-up time. Thus, the HIV mortality risk model from previous studies (Hou et al., 2019; Wang et al., 2020) was used to predict HIV/AIDS-related mortality of RI and LI patients. The results indicated that the survival probability of the RI group was 98.69%, 98.20%, and 97.68% at the 1st, 2nd and 3rd year after cART, respectively, which were higher than the LI group (96.77% at the 1st year; 95.56% at the 2nd year; 94.30% at the 3rd year, *p* < 0.001, Figures 5A,B). Meanwhile, similar results were obtained when both age and gender were adjusted. Further analysis suggested that the survival probability of LI AIDS patients was significantly smaller than RI and LI non-AIDS patients (*p* < 0.001, Figures 5C,D). The prognostic risk scores indicated that 22.22% of RI patients belong to the moderate risk group while 34.41% were in the LI non-AIDS group and 98.33% in the LI AIDS group (*p* < 0.001, Figure 6). These results showed lower HIV/AIDS-related mortality risk in RI patients than LI patients.

## Discussion

In this study, we developed a rapid HIV-1 recency test to distinguish HIV-1 RI or LI patients and further assessed the impact of differentiating HIV-1 RI and LI on virological suppression and rebound, immune reconstitution as well as mortality after cART. We demonstrated the good performance of our rapid HIV-1 recency testing RRITS and excellent consistency with the commercial LAg-Avidity EIA assay in detecting both longitudinal and cross-sectional samples. These results proved the feasibility of implementing rapid HIV-1 recency testing assay to determine the status of HIV-1 patients in clinics, hospitals and and in resource-limiting settings.

HIV-1 recency test can be used to estimate HIV-1 incidence in the population since the ordinary anti-HIV immunoassay can only diagnose HIV-1 infection and determine the prevalence of HIV-1. Currently, several assays can distinguish HIV-1 RI and LI and can be used to estimate HIV-1 incidence, such as Western blot and the Fiebig staging method (Fiebig et al., 2003), LAg-Avidity EIA and BED-CEIA method (Le et al., 2013). We have previously described an immunosorbent assay to distinguish HIV-1 RI and LI (Li et al., 2016; Cai et al., 2019). In this study, we further refined the assay format to develop a rapid immunoassay HIV-1 RRITS for POCT. In contrast, the widely used BED-CEIA and LAg-Avidity EIA have stringent criteria for experimental equipment and experimental operators, as well as dozens of testing steps and several hours of experimental time; hence, they are unsuitable for rapid on-site testing. Compared with the current methods, the main advantages of RRITS are as follows: (1) the operation of RRITS is straightforward and does not need experimental equipment; (2) the sample tested does not require dilution; (3) the experiment only takes 30 min; (4) HIV-1 infection diagnosis and differentiation of HIV-1 RI and LI can be performed simultaneously.

When the HIV-1 recency test was used to determine the stage of HIV-infected individuals, the accuracy and reliability are still debated. Therefore, the UNAIDS recommends the RITAs by combining HIV-1 serological recency testing with clinical tests to accurately distinguish RI and LI (WHO, 2011; accessed January 23, 2022). It has been reported that excluding cases with CD4^+^ T-cell count <200 cells/μl and viral load <1,000 copies/ml can reduce FRR from 4.9% to 0 (Karatzas-Delgado et al., 2020). Currently, CD4^+^ T-cell count test is provided for free to HIV-infected individuals in China, and HIV-1 viral load testing has been used since 2019. Thus, integrating the history of HIV-1 diagnosis, CD4^+^ T-cell count and HIV-1 RNA level and HIV-1 recency testing results into RITAs for accurate classification of HIV-1 infection status is feasible in China and provides us an opportunity to assess the role of RITAs in managing cART-treated patients. Based on RITAs and our rapid HIV-1 recency test, we found that in our study, only 22.5% of HIV-infected subjects were in the stage of HIV-1 recent infection while 77.5% of them were chronic infection and 30% of them have been AIDS patients when they started cART. Our results indicated that early diagnosis and initiation of cART are still an important but unresolved issue in China.

Furthermore, we found that 4.1% of LI patients experienced virological rebound in the 1st year of cART and none of the RI patients relapsed, suggesting that RI patients were able to achieve and maintain more stable virological suppression after receiving cART, which was consistent with the previous studies (Jain et al., 2013; Buzon et al., 2014). The cause of virological rebound is complicated. It is important to effectively control and reduce the viral reservoir to decrease virological rebound (Deeks et al., 2021). Although cART can effectively inhibit viral replication and viral loads, but cannot eradicate HIV-1 since HIV-1 DNA can still be detected in CD4^+^ T-cell in the blood and lymphoid tissues of cART-treated HIV-1 patients. Previous studies have found that starting cART in the acute phase of HIV-1 infection can accelerate the attenuation of the viral reservoir and maintain a lower and more stable virological suppression (Hocqueloux et al., 2013; Laanani et al., 2015), and also promotes the immune response of HIV-specific T helper cells, and hinders the establishment and expansion of viral reservoirs (Lori et al., 1999; Ananworanich et al., 2012; Archin et al., 2012). Several studies have shown that early cART can reduce microbial translocation, immune activation level and lymphoid tissue damage, and promote immune recovery of HIV-infected patients (Brenchley and Douek, 2008; Buzón et al., 2010; Rajasuriar et al., 2010; Fernandez et al., 2011; Hunt, 2012; Zeng et al., 2012a,b). Previous studies have shown that early cART among the recently HIV-infected patients can quickly eliminate lymphoid tissue damage caused by HIV-1, and enhance the recovery of CD4^+^ T-cell (Zeng et al., 2012a,b). Even under sustained virological suppression, 30% of cART-treated HIV-infected patients showed abnormal CD4^+^ T-cell count (Battegay et al., 2006; Gazzola et al., 2009; Handoko et al., 2020). Low CD4^+^ T-cell count increases the risk of morbidity and mortality of AIDS and non-AIDS-related complications (Baker et al., 2008; Al-Mrabeh et al., 2016) even though these patients have reached virological suppression (Marin et al., 2009; Belloso et al., 2010). One of the reasons for this phenomenon may be that the immune function of LI patients was impaired, which affects the recovery of CD4^+^ T-cell. Our study also showed that 45.2% of HIV-1 patients did not reach the CD4^+^ T-cell count of 500 cells/mm^3^ after 2 years of cART and 95.7% of them were LI patients. Under cART, CD4^+^ T-cell count was always higher in the RI group than in the LI group.

It is reported that CD4/CD8 ratio is a reliable marker of systemic immune activation for HIV-infected patients under cART (Buggert et al., 2014; Bruno et al., 2017). Although HIV-infected patients with virological suppression and CD4^+^ T-cell count ≥500/mm^3^, a low CD4/CD8 ratio is also associated with immune failure and abnormal activation, e.g., monocyte activation (Serrano-Villar et al., 2014; Lu et al., 2015). In addition, several studies have shown that CD4/CD8 ratio inversion and high CD8^+^ T-cell count (≥2,000/mm^3^) can increase the risk of all-cause mortality of HIV-1 patients despite receiving effective cART (Mussini et al., 2015; Sigel et al., 2017; Trickey et al., 2017; Caby et al., 2021). The SPARTAC and UK trials indicated that CD4/CD8 ratios recovered to normal (≥1) in 45.1% of recently infected HIV-1 patients after 1 year of cART, but only 11.1% for the patients with delayed cART (Fidler et al., 2013; Thornhill et al., 2016). In our study, we found that the probability of CD4/CD8 ratio recovering to 1 was 3.6 times higher in RI patients than LI patients, which may be caused by the continuous inversion of the CD4/CD8 ratio due to the excessive activation of CD8^+^ T-cell and the slow recovery speed of CD4^+^T-cell in LI patients (Caby et al., 2021).

Although the survival of HIV-1 patients has been greatly extended with the widespread use of cART, LI patients may still face greater prognostic risk. In our study, the HIV/AIDS-related mortality risk was lower in RI patients than LI patients. It has been reported that the possibility of HIV/AIDS-related death was 20 times higher in those with moderate prognostic risk than in those with low prognostic risk (Hou et al., 2019). In our study, we found that 22.22% of RI patients had moderate prognostic risk scores, which was lower than 34.41% in the LI non-AIDS patients group and 98.33% in the LI AIDS group. These results also support the higher risk of HIV/AIDS-related death in LI patients even under cART therapy.

Our study has several limitations. First, more HIV-1 infection specimens with seroconversion dates need to be collected to accurately assess FRR and MDRI of the HIV-1 recency test. Second, few clinical endpoints have been observed due to the relatively short observation period. The clinical cohort study is still ongoing. Third, we did not measure HIV-1 DNA levels to evaluate the dynamic of HIV-1 viral reservoir in RI and LI patients after initiation of cART. Finally, we would like to emphasize that HIV-1 recency test may be used as an aid for the management of HIV-1 patients for clinical use, but not for determining the stage of HIV-1 infection for individuals.

In conclusion, we successfully developed a rapid HIV-1 recency test, which can be feasibly implemented in point-of-care settings. Our study supports the incorporation of rapid HIV-1 recency test into RITAs to accurately distinguish HIV-1 RI and LI and to improve the management of HIV-1 patients. Of note, we found that a rapid HIV-1 recency test alone could achieve similar results of differentiating HIV-1 RI and LI as RITAs, suggesting that in the resource-limiting settings, rapid HIV-1 recency test alone may help monitor HIV-1 incidence and predict the prognosis of HIV-1 patients.

## Data availability statement

The raw data supporting the conclusions of this article will be made available by the authors, without undue reservation.

## Ethics statement

The studies involving human participants were reviewed and approved by Nanfang Hospital Ethics Committee; Nanfang Hospital of Southern Medical University. The patients/participants provided their written informed consent to participate in this study.

## Author contributions

JZ: analysis and interpretation of data, conduction of experiment, and drafting of the manuscript. HC and TY: acquisition of data, analysis and interpretation of data. ZW: performed experiment. QL: performed the data analysis, figures plotted. JS and HW: revision of the manuscript. ST and JP: conception, design, and finalizing of the manuscript. All authors contributed to the article and approved the submitted version.

## Funding

This work was supported by the National Major Science and Technology Project (grant number 2018ZX10302103-002) and Bureau of Science and Information Technology of Guangzhou Municipality (grant no. 201704020219).

## Conflict of interest

The authors declare that the research was conducted in the absence of any commercial or financial relationships that could be construed as a potential conflict of interest.

The handling editor declared a past co-authorship with the author [ST].

## Publisher’s note

All claims expressed in this article are solely those of the authors and do not necessarily represent those of their affiliated organizations, or those of the publisher, the editors and the reviewers. Any product that may be evaluated in this article, or claim that may be made by its manufacturer, is not guaranteed or endorsed by the publisher.

## Supplementary material

The Supplementary material for this article can be found online at: https://www.frontiersin.org/articles/10.3389/fmicb.2022.1004960/full#supplementary-material

Supplementary Figure S1 Determination of volume ratio of the BE23 and MP4 antigen (Ag) at the T_1_ testing line of HIV-1 rapid recent-infection testing strip (RRITS). The concentrations of the BE23 and MP4 were 0.30 mg/ml and 0.45 mg/ml, respectively.

Supplementary Figure S2 Determination of cut-off value of T_1_ testing line based on receiver operator characteristic curve (ROC) and maximal Youden index.

Supplementary Figure S3 The scatter diagram between the T_1_ testing line score of HIV-1 rapid recent-infection testing strip (RRITS) and ODn of LAg-Avidity EIA using specimens, including longitudinal and cross-sectional samples (n = 431).

Supplementary Figure S4 Rapid recent-infection testing strip (RRITS) alone and recent infection testing algorithms (RITAs) based on LAg-Avidity EIA to distinguish recent and long-term HIV-1 infection. LS_T1_, the T_1_ testing line score of RRITS; HIV-1, human immunodeficiency virus I; AIDS, acquired immunodeficiency syndrome; VL, viral load.

Supplementary Figure S5 The recovery speeds of CD4^+^ T-cell count (A) and CD4/CD8 ratio (B) among HIV-1 recent infection and long-term infection with or without AIDS groups during 12 months of cART. *, *p* < 0.05; **, *p* < 0.01; ***, *p* < 0.001; ns, *p* > 0.05. RI, recent infection; LI, long-term infection; mo, month.

Supplementary Figure S6 The recovery speeds of CD4^+^ T-cell count (A) and CD4/CD8 ratio (B) in all, recent infection and long-term infection patients during consecutive time intervals after initiation of cART. *, *p* < 0.05; **, *p* < 0.01; ***, *p* < 0.001; ****, *p* < 0.0001; ns, *p* > 0.05: RI, recent infection; LI, long-term infection; mo, month.
