# Supplementary figures and images for: Evaluation of antiretroviral therapy effect and prognosis between HIV-1 recent and long-term infection based on a rapid recent infection testing algorithm
Source: Front Microbiol. 2022 Nov 22;13:1004960. doi: 10.3389/fmicb.2022.1004960 (PMC9722761; doi:10.3389/fmicb.2022.1004960)

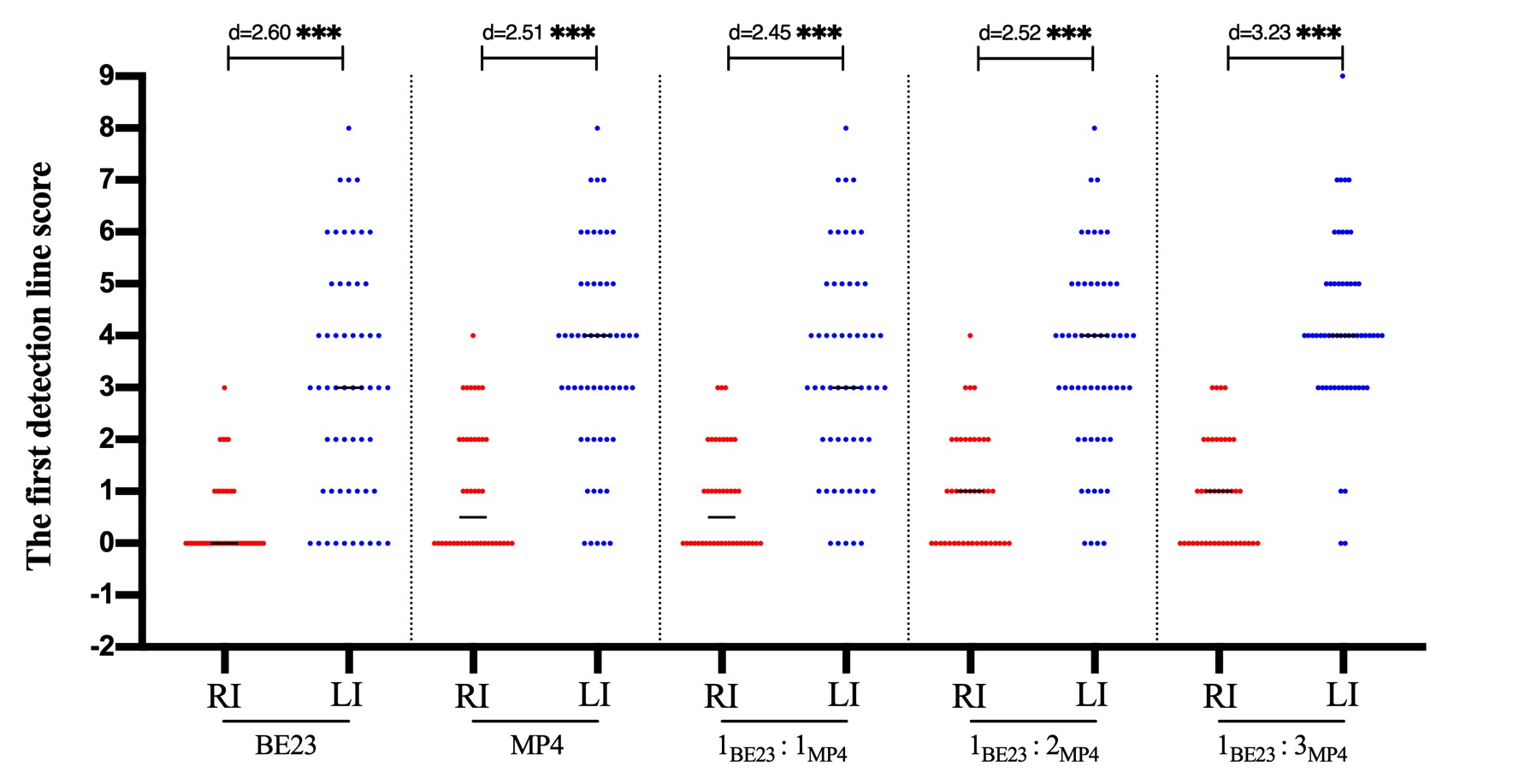

Supplement: Supplementary Figure S1 — Determination of volume ratio of the BE23 and MP4 antigen (Ag) at the T1 testing line of HIV-1 rapid recent-infection testing strip (RRITS). The concentrations of the BE23 and MP4 were 0.30 mg/ml and 0.45 mg/ml, respectively. [file Image_1.JPEG]

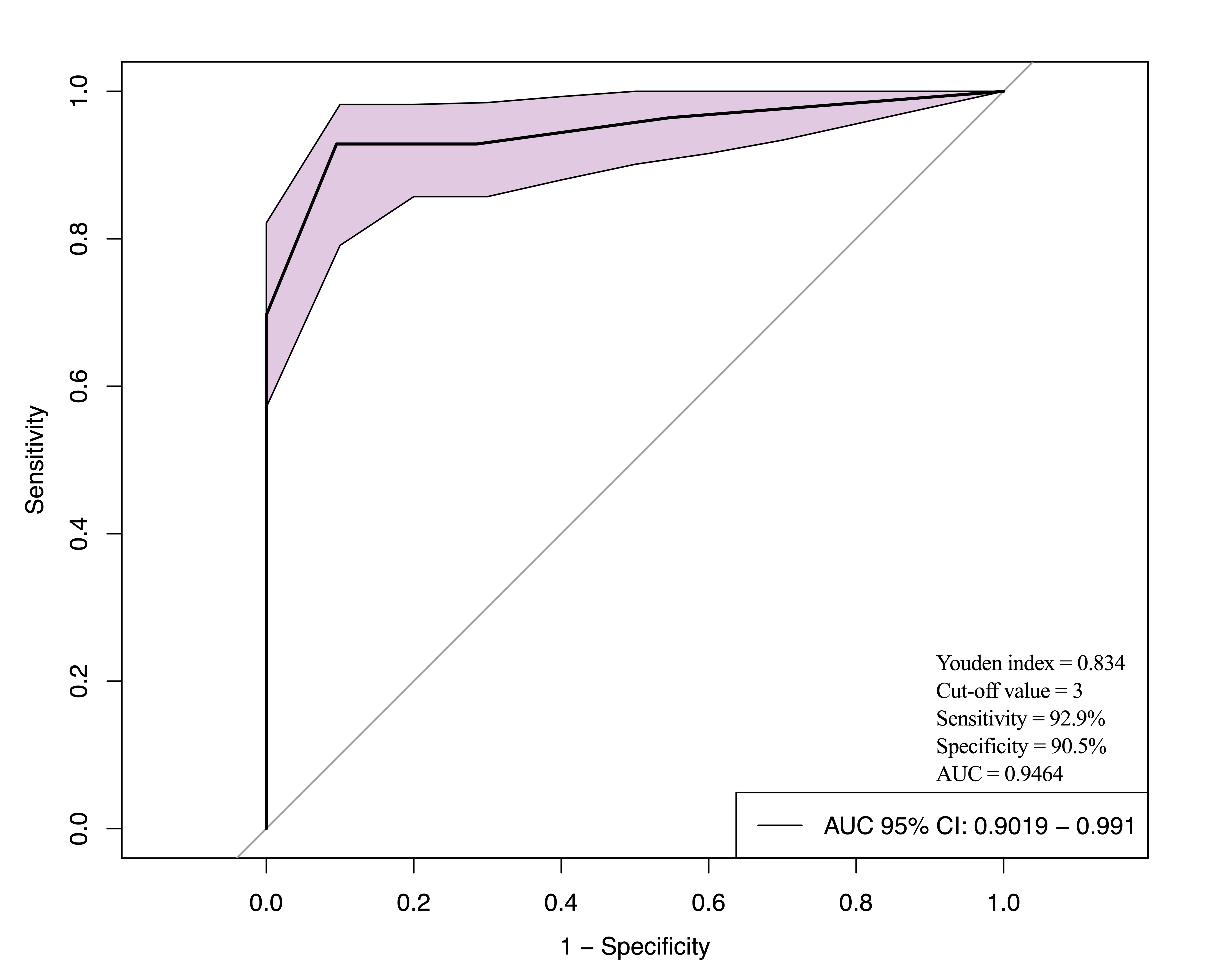

Supplement: Supplementary Figure S2 — Determination of cut-off value of T1 testing line based on receiver operator characteristic curve (ROC) and maximal Youden index. [file Image_2.JPEG]

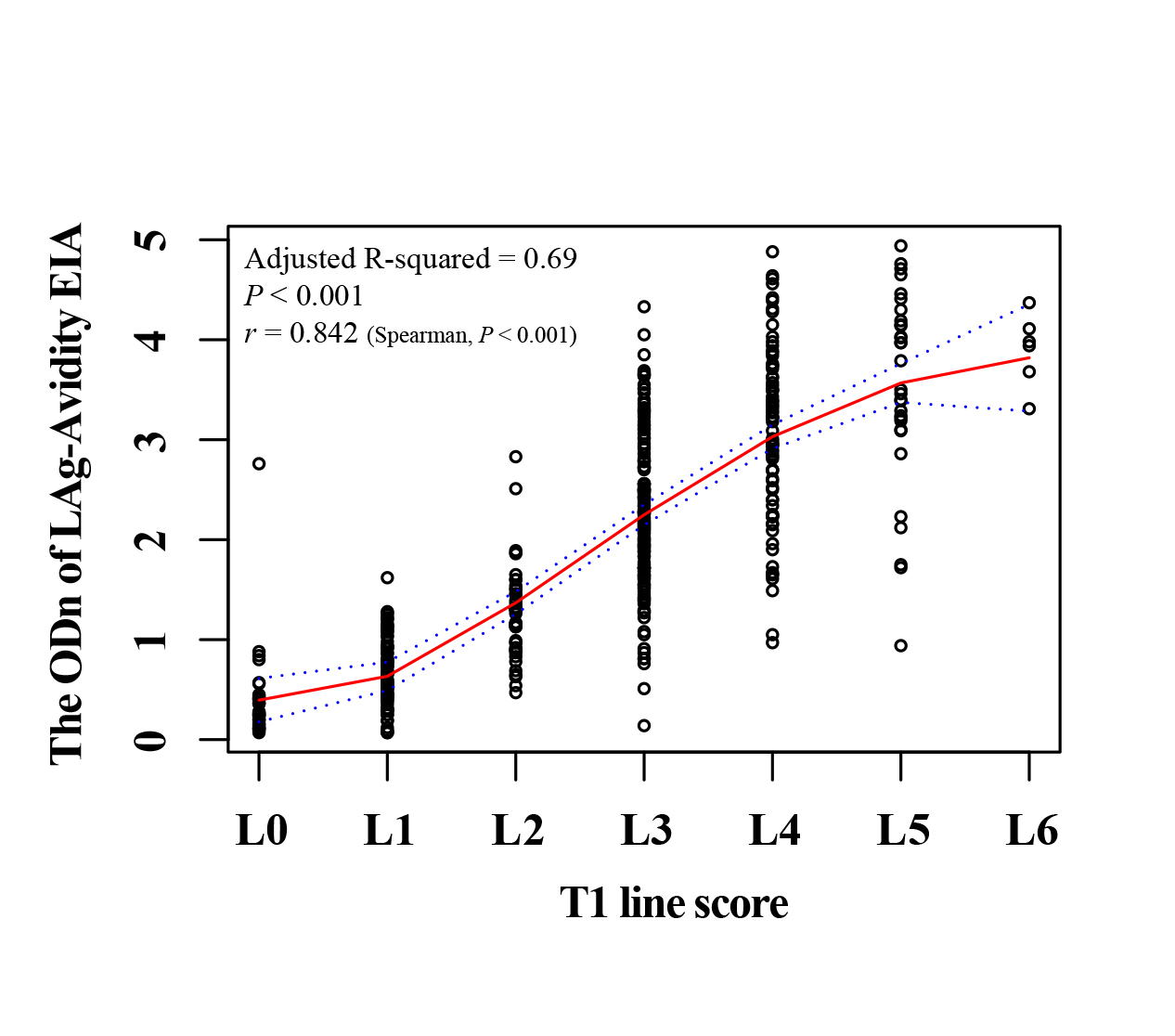

Supplement: Supplementary Figure S3 — The scatter diagram between the T1 testing line score of HIV-1 rapid recent-infection testing strip (RRITS) and ODn of LAg-Avidity EIA using specimens, including longitudinal and cross-sectional samples (n = 431). [file Image_3.JPEG]

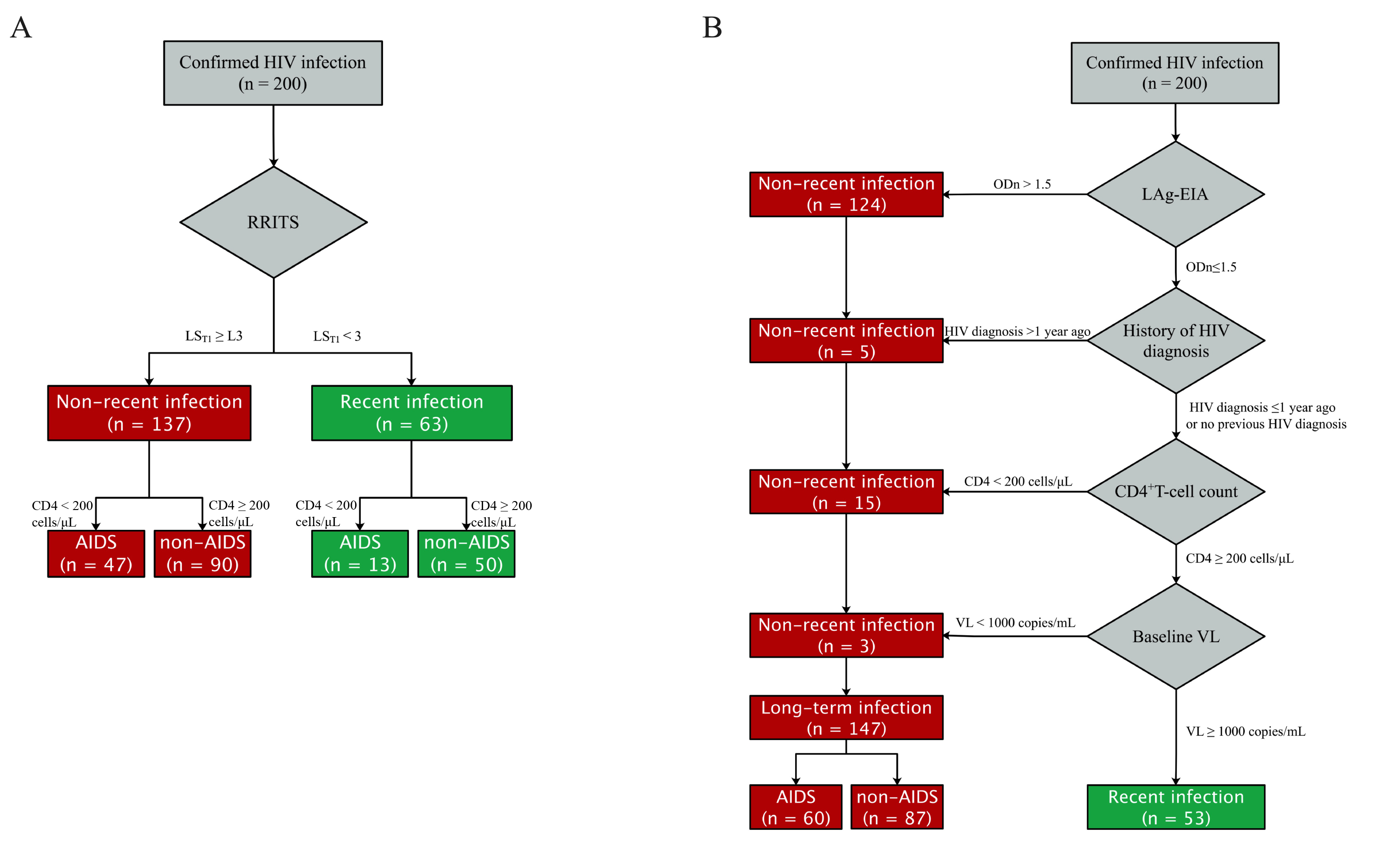

Supplement: Supplementary Figure S4 — Rapid recent-infection testing strip (RRITS) alone and recent infection testing algorithms (RITAs) based on LAg-Avidity EIA to distinguish recent and long-term HIV-1 infection. LST1, the T1 testing line score of RRITS; HIV-1, human immunodeficiency virus I; AIDS, acquired immunodeficiency syndrome; VL, viral load. [file Image_4.JPEG]

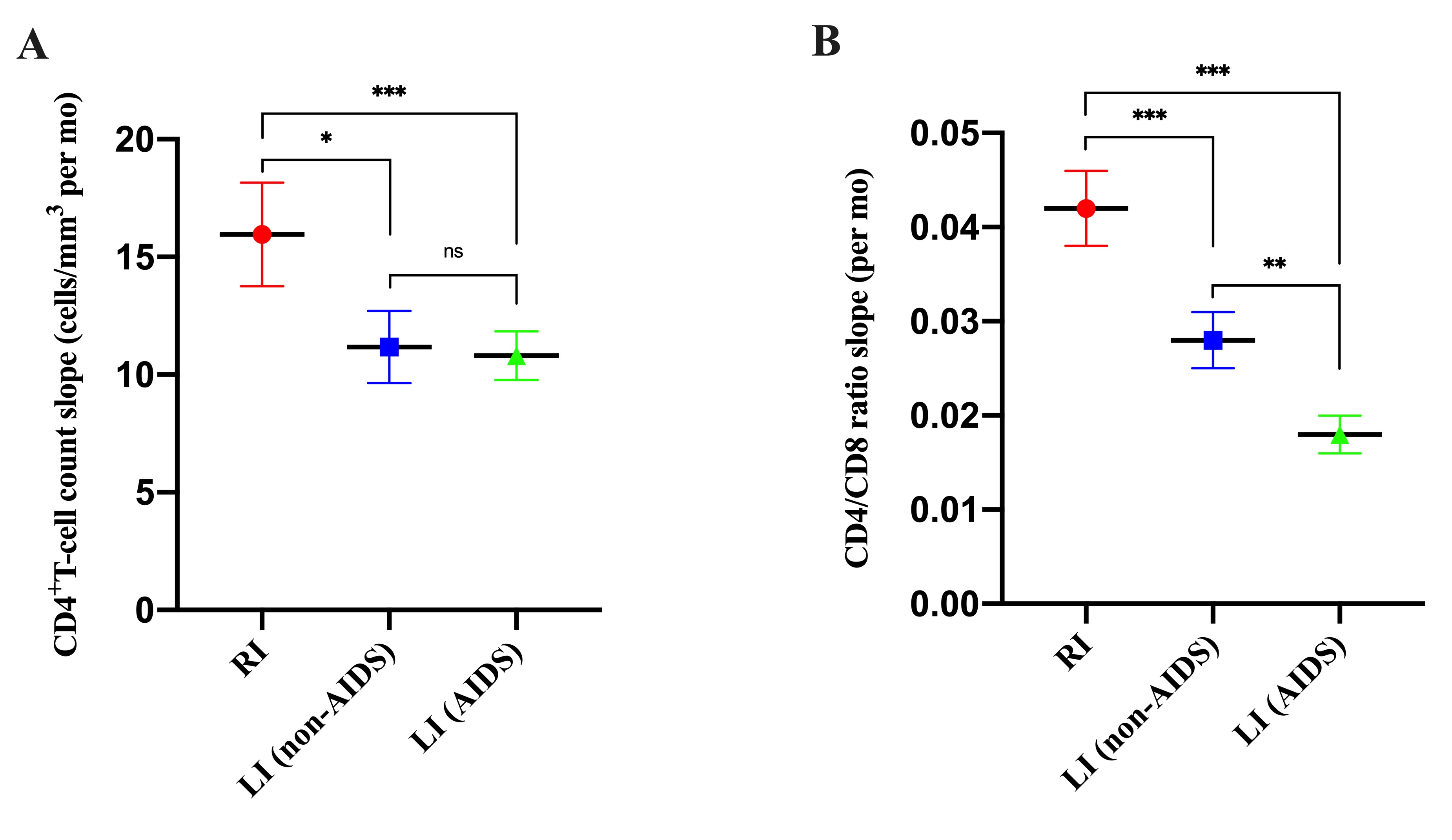

Supplement: Supplementary Figure S5 — The recovery speeds of CD4+ T-cell count (A) and CD4/CD8 ratio (B) among HIV-1 recent infection and long-term infection with or without AIDS groups during 12 months of cART. *, p < 0.05; **, p < 0.01; ***, p < 0.001; ns, p > 0.05. RI, recent infection; LI, long-term infection; mo, month. [file Image_5.JPEG]

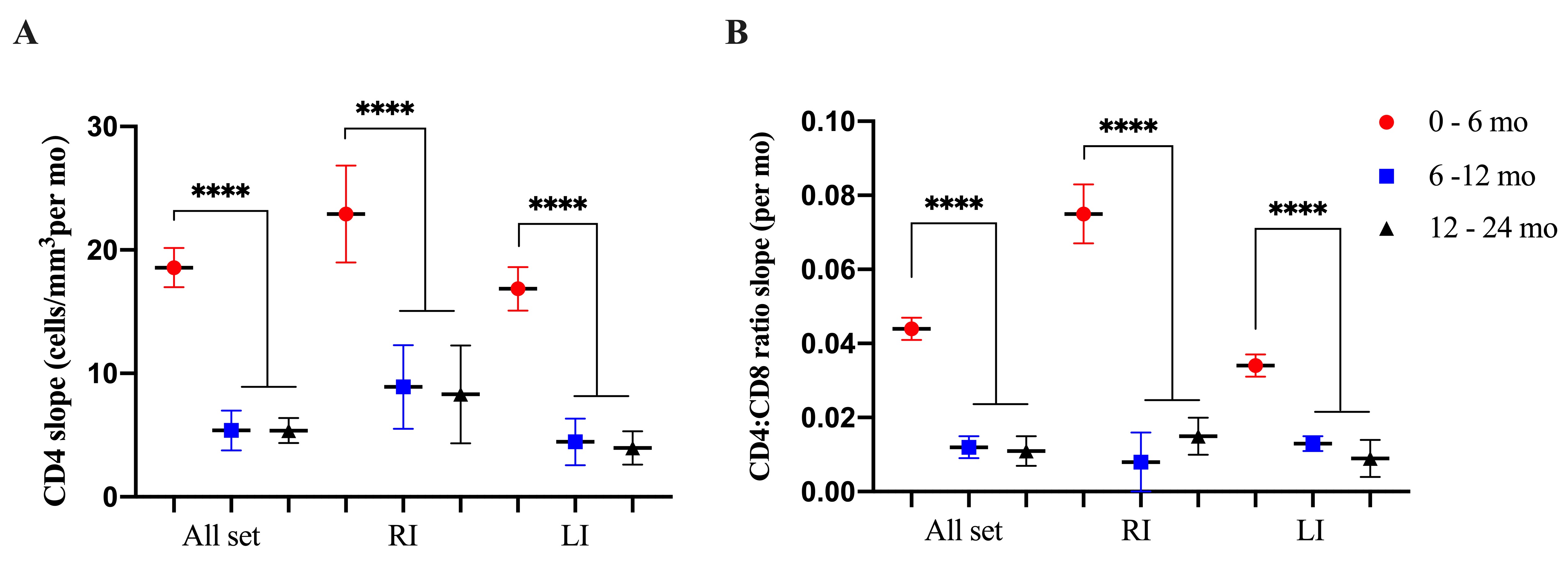

Supplement: Supplementary Figure S6 — The recovery speeds of CD4+ T-cell count (A) and CD4/CD8 ratio (B) in all, recent infection and long-term infection patients during consecutive time intervals after initiation of cART. *, p < 0.05; **, p < 0.01; ***, p < 0.001; ****, p < 0.0001; ns, p > 0.05: RI, recent infection; LI, long-term infection; mo, month. [file Image_6.JPEG]
