# Supplementary material for: Evaluation of antiretroviral therapy effect and prognosis between HIV-1 recent and long-term infection based on a rapid recent infection testing algorithm
Source: Front Microbiol. 2022 Nov 22;13:1004960. doi: 10.3389/fmicb.2022.1004960 (PMC9722761; doi:10.3389/fmicb.2022.1004960)
Supplement: Supplementary file 7 [file Table_1.DOCX]

**Table S1.** Specimen panels in this study.

| Specimen panel | Institution | Component |
| --- | --- | --- |
| Panel 1 | (1) Beijing Xinchuang  Bioengineering Co., Ltd  (2) Guangzhou Blood Center | (1) 98 HIV-1 archived samples (11 CRF01_AE, 31 CRF07_BC, 14 CRF08_BC, 3 Subtype B, 39 Unknow)  (2) 20 HIV-1 anti-body negative samples (Healthy volunteers) |
| Panel 2 | Archived de-linked | 36 HIV-1 longitudinal samples |
| Panel 3 | (1) Guangzhou Center for Disease Control and Prevention  (2) The Eighth People's Hospital of Guangzhou  (3) Nanfang Hospital of Guangdong | (1) 110 HIV-1 cross-sectional samples (40 CRF01_AE, 40 CRF_07BC, 7 Subtype B and 23 CRF55_01B)  (2) 85 HIV-1 cross-sectional samples  (3) 200 HIV-1 cohort samples |

**Table S2.** Comparison between HIV-1 RRITS and anti-HIV enzyme-linked immunosorbent assay (ELISA) for detecting anti-HIV-1 antibody.

| Methods | | Anti-HIV ELISA | | | Sensitivity | Specificity | *P*-value ^a^ | Kappa |
| --- | --- | --- | --- | --- | --- | --- | --- | --- |
|  |  | + | - | Total |  |  |  |  |
| RRITS | + | 97 | 0 | 97 | 98.98% | 100.00% | > 0.999 | 0.96 |
|  | - | 1 | 20 | 21 |  |  |  |  |
|  | Total | 98 | 20 | 118 |  |  |  |  |

^a^ *P* value was calculated by using the McNemar Test to analyze whether the positive rates of the two immunoassays were consistent.

ELISA, enzyme-linked immunosorbent assay.

**Table S3** Results of HIV-1 RRITS for different HIV-1 subtype samples

| Subtype | RRITS | LAg-Avidity EIA | | Total | *P-value*^a^ | κ ^b^ | Sensitivity  (%) | Specificity (%) |
| --- | --- | --- | --- | --- | --- | --- | --- | --- |
|  |  | RI | LI |  |  |  |  |  |
| CRF01_AE | RI | 17 | 2 | 19 | >0.999 | 0.80 | 89.47 | 90.48 |
|  | LI | 2 | 19 | 21 |  |  |  |  |
|  | Total | 19 | 21 | 40 |  |  |  |  |
| Non-CRF01_AE | RI | 20 | 2 | 22 | >0.999 | 0.84 | 86.96 | 95.74 |
|  | LI | 3 | 45 | 48 |  |  |  |  |
|  | Total | 23 | 47 | 70 |  |  |  |  |
| CRF07_BC | RI | 10 | 1 | 11 | >0.999 | 0.82 | 83.33 | 96.43 |
|  | LI | 2 | 27 | 29 |  |  |  |  |
|  | Total | 12 | 28 | 40 |  |  |  |  |
| Non-CRF07_BC | RI | 27 | 3 | 30 | >0.999 | 0.83 | 90.00 | 92.50 |
|  | LI | 3 | 37 | 40 |  |  |  |  |
|  | Total | 30 | 40 | 70 |  |  |  |  |
| Subtype B | RI | 2 | 0 | 2 | >0.999 | NT ^c^ | 100.00 | 100.00 |
|  | LI | 0 | 5 | 5 |  |  |  |  |
|  | Total | 2 | 5 | 7 |  |  |  |  |
| Non-B | RI | 35 | 4 | 39 | >0.999 | 0.82 | 87.50 | 93.65 |
|  | LI | 5 | 59 | 64 |  |  |  |  |
|  | Total | 40 | 63 | 103 |  |  |  |  |
| CRF5501_B | RI | 8 | 1 | 9 | >0.999 | 0.82 | 88.89 | 92.86 |
|  | LI | 1 | 13 | 14 |  |  |  |  |
|  | Total | 9 | 14 | 23 |  |  |  |  |
| Non-CRF5501_B | RI | 29 | 3 | 32 | >0.999 | 0.83 | 87.88 | 94.44 |
|  | LI | 4 | 51 | 55 |  |  |  |  |
|  | Total | 33 | 54 | 87 |  |  |  |  |

^a^ *P-value* was calculated by McNemar

^b^ κ, Kappa

^c^ NT, κ cannot be calculated

Abbreviation: RRITS, Rapid recent-infection testing strip; RI, recent infection; LI, long-term infection.

**Table S4.** Demographic and clinical characteristics between HIV-1 recent infection and long-term infection groups after propensity score matching ^a^.

| RITAs | RRITS | | Total | *P*-value ^a^ | Kappa |
| --- | --- | --- | --- | --- | --- |
|  | RI | LI |  |  |  |
| RI | 45 | 0 | 45 | <0.01 | 0.774 |
| LI | 18 | 137 | 155 |  |  |
| Total | 63 | 137 | 200 |  |  |

^a^ *P*-value was calculated by McNemar's test.

Abbreviation: RRITS, Rapid recent-infection testing strip; RITAs, recent infection testing algorithms; RI, recent infection; LI, long-term infection.

**Table S5.** Demographic and clinical characteristics between HIV-1 recent infection and long-term infection groups after propensity score matching ^a^.

| **Variables** | **RI**  (n=35) | **LI**  (n=35) | **Total**  (n=70) | ***P*-value** ^b^ |
| --- | --- | --- | --- | --- |
| **Age** (year), median (IQR) | 25.0  (22.0, 35.0) | 25.0  (23.0, 29.0) | 25.0  (22.0, 31.0) | 0.981 |
| **Baseline CD4^+^ T-cell count** (cells/mm^3^), median (IQR) | 381.0  (291.0, 448.0) | 378.0  (235.0, 503.0) | 379.5  (281.8, 467.8) | 0.851 |
| **Baseline HIV-1 RNA level** (log_10_ copies/mL), median (IQR) | 4.27  (3.75, 4.84) | 4.20  (3.72, 4.81) | 4.24  (3.74, 4.82) | 0.626 |
| **Gender**, no. (%) |  |  |  | >0.999 |
| Female | 1 (2.9) | 0 (0) | 1 (1.4) |  |
| Male | 34 (97.1) | 35 (100) | 69 (98.6) |  |
| **Mode of infection,** no. (%) |  |  |  | >0.999 |
| Heterosexual contact | 3 (8.6) | 2 (5.7) | 5 (7.1) |  |
| Homosexual contact | 32 (91.4) | 33 (94.3) | 65 (92.9) |  |
| **Marital status**, no. (%) |  |  |  | 0.511 |
| Married or cohabiting | 7 (20) | 5 (14.3) | 12 (17.1) |  |
| Divorced or separated or widowed | 3 (8.6) | 1 (2.9) | 4 (5.7) |  |
| Unmarried | 25 (71.4) | 29 (82.9) | 54 (77.1) |  |
| **cART regimen**, no. (%) |  |  |  | >0.999 |
| TDF + 3TC+ EFV | 33 (94.3) | 32 (91.4) | 65 (92.9) |  |
| TDF+ 3TC+ LPV/r | 0 (0) | 1 (2.9) | 1 (1.4) |  |
| TDF+ 3TC+ DTG | 1 (2.9) | 0 (0) | 1 (1.4) |  |
| E+ C+ T+ F | 1 (2.9) | 2 (5.7) | 3 (4.3) |  |
| **cART compliance**, no. (%) |  |  |  | 0.403 |
| Optimal | 25 (71.4) | 28 (80) | 53 (75.7) |  |
| Sub-optimal | 10 (28.6) | 7 (20) | 17 (24.3) |  |

^a^ Propensity score matching was used to conduct a 1:1 case-control study by controlling age, baseline CD4^+^ T cell count, baseline HIV-1 RNA level, gender, mode of infection, marital status, cART regimen and drug therapy compliance.

^b^ Comparison between groups before cART: Mann-Whitney test, or χ^2^ test or Fisher exact test.

Abbreviation: RI, recent infection; LI, long-term infection; TDF, tenofovir disoproxil fumarate; 3TC, lamivudine; EFV, efavirenz; LPV/r, lopinavir/ritonavir; E, elvitegravir; C, cobicistat; T, tenofovir; F, emtricitabine; cART, combination antiretroviral therapy; IQR, interquartile rang.
