# Supplementary material for: Evaluation of antiretroviral therapy effect and prognosis between HIV-1 recent and long-term infection based on a rapid recent infection testing algorithm
Source: Front Microbiol. 2022 Nov 22;13:1004960. doi: 10.3389/fmicb.2022.1004960 (PMC9722761; doi:10.3389/fmicb.2022.1004960)
Supplement: Supplementary file 8 [file Presentation_1.PDF]

Materials and methods of developing HIV-1 rapid recent-infection testing strip (RRITS)

Assembly of HIV-1 RRITS

The colloidal gold-labeled mouse anti-human IgG antibody, sheep anti-mouse IgG and HIV-1 recombinant antigen p101 (88) (XM1008HIV-A) were purchased from the company InTec (Xiamen, China). HIV-1 gp41 recombinant antigen BE23 and MP4 were expressed and purified in InTec and our lab, respectively. The amino acid sequences of BE23 and MP4 were shown in Table 1. The lateral flow device of the RRITS was assembled with a sample application pad, conjugate pad, nitrocellulose membrane, and wicking pad purchased from InTec (Xiamen, China, Figure 1). The mouse anti-human IgG was combined with colloidal gold particles to form a colloidal gold-labeled mouse anti-human IgG antibody, and evenly coated on the conjugate pad with a concentration of 10-20 μg/mL, then dried at 45°C. The nitrocellulose membrane was coated with the recombinant antigens BE23 and MP4, HIV-1 antigen 101, and goat anti-mouse antibody to represent the first detection line (T_1), the second detection line (T_2), and control line (T_c), respectively, and dried for 18-22h at 25°C and humidity of 10-30%. The specific HIV-1 gp41 immunodominant epitopes (IDEs) in recombinant antigens BE23 and MP4 could differentiate recent and long-term HIV-1 infections based on the affinity of antibodies at different levels of maturation. Moreover, ability to discriminate that MP4 benefit from two major IDEs of GKIIC and QKFLG motifs located in the loop region of HIV-1 gp41 and the upstream and downstream amino acid

residues of these IDEs (Cai et al., 2019). The T_1 consists of HIV-1 gp41 recombinant antigen BE23 and MP4 that block the binding of anti-gp41 antibody in particular for the immature and low avidity anti-HIV antibody from HIV-1 recently infected subjects. The mature antibodies in long-term HIV infections could bind HIV-1 gp41 recombinant antigens BE23 and MP4 to make T1 color, while not in recent HIV infection infections. The work concentration was 3.5 mg/mL for HIV-1 recombinant antigen P101, 0.3 mg/mL for gp41 recombinant antigen BE23 and 0.45 mg/mL for MP4. The work concentration of goat anti-mouse IgG was 1.0 mg/mL. The sample application pad, conjugate pad, nitrocellulose membrane, and wicking pad were assembled in order and cut into 5 mm strips stored at relative humidity < 25% before use.

Operation of HIV-1 RRITS

For HIV-1 recency testing, 10uL specimen and 3-4 drops (100-150 uL) of sample dilution buffer phosphate-buffered saline (PBS) were dropped on the sample application pad. The results of RRITS were read after 30min at room temperature (25°C). The intensity of three detection lines was independently scored by two persons using a standard color card (L0-L10).

The interpretation of HIV-1 RRITS results

The results of test strips were compared with standard color card. Firstly, the quality control line Tc should be greater than L0, then the experimental results are valid. Secondly, T_2 test line should be greater than L0 that showed the samples tested in this

experiment were HIV-1 positive samples. On the basis of satisfying the above premises, T1 test line score of samples ≥ L3 is identified as HIV-1 long-term infection, while T1 test line score of samples < L3 is identified as HIV-1 recent infection. The detailed algorithm used for the interpretation of testing results is shown in Figure 1B-1D.

Specimen panels

The optimization of our HIV-1 recency testing RRITS was performed using specimens of Panel One that contains 118 samples including 98 anti-HIV positive and 20 anti-HIV negative healthy volunteer samples obtained from Beijing Xinchuang Bioengineering Co., Ltd (Beijing, China) and detected by anti-HIV enzyme-linked immunosorbent assay (ELISA) of WANTAI BioPharm (Beijing, China). HIV-1 positive samples were further classified into HIV-1 RI and LI groups by using the commercial BED-CEIA (Sedia Biosciences, Portland, OR, USA) and LAg-Avidity EIA (Kinghawk Pharmaceutical Co., Ltd, Beijing, China) kits. Panel 2 included 36 archived de-linked serum samples from 9 patients undergoing acute HIV-1 seroconversion. They were prospectively collected and have been previously described (Cai et al., 2019), and were used to calculate the mean duration of recent infection (MDRI) by polynomial regression. In theory, the mean infection time of HIV-1 LI patients differentiated by RRITS was more than MDRI. Each patient provided 4 prospective samples from the last date with anti-HIV negative result (day 0) up to 602 days. Panel 3 included 110 specimens from the Guangzhou Center for Disease Control and Prevention, 200 from Nanfang Hospital of Guangzhou and 85 from the Guangzhou Eighth People's Hospital.

These samples were detected by the recency test of HIV-1 LAg-Avidity EIA Kit and used to further validate the performance of our HIV-1 RRITS. The detailed information for Panels 1-3 were shown in Table S2. 34 HIV-1 serum samples for calculating false-recent rate (FRR) of RRITS that had been infected for more than 1 year and were not receiving cART at the time of serum sample collection were included, including 11 archived de-linked serum samples and 23 serum samples from the HIV-1 cohort of Nanfang Hospital before starting cART. FRR formula: $FRR = \frac{\text{false recent } cases * 100\%}{\text{total recent cases (the duration of infection } > 1 \text{ } year)}$.

Reference

Cai, Q., Wang, H., Huang, L., Yan, H., Zhu, W., and Tang, S. (2019). Characterization of HIV-1 genotype specific antigens for the detection of recent and long-term HIV-1 infection in China. *Virus research* 264**,** 16-21. doi: 10.1016/j.virusres.2019.02.010.

Table 1. Amino acid sequences of gp41 recombinant antigen BE23 and MP4.

Antigen name	Sequences
BE23	metseraspl ysileilehi sleuthrasp aspserphea spthraspva lleulysala aspglyalai leleuvalas pphetrpala glutrpcysg lyprocysly smetileala proileleua spgluileal aaspglutyr glnglylysl euthrvalal alysleuasn ileaspglna snproglyth ralaprolys tyrglyilea rgglyilepr othrleuleu leuphelysa snglygluva lalaalathr lysvalglya laleuserly sglyglnleu lysgluphel euaspalaas nleualamet glyleuglna laargvalle ualavalglu argtyrleul ysaspglnly spheleugly leutrpglyc ysserglyly sileilecys thrthralaa laprogluph earrglyasp lysaspglyg lyleuglnal aargileleu alaileglua rgtyrleugl naspglngln leuleuglyi letrpglycy sserglylys hisilecyst hrthrthrly sleuarggly asplysaspg lyglyleugl nalaargile leualavalg luargtyrle ulysaspgln glnleuleug lyiletrpgl ycysserglу lysleuilec ysthrthrth ralapro
MP4	glualaglng lnhisleule uglnleuthr valtrpglyi lelysglnle uglnalaarg valleualav algluargty rleulysasp glnglnleul euglyiletr pglycysser glylysleui lecysthrth ralavalpro trpasnalas ertrpseras nglyglygly glyserglyg lyglyglyse rglualagln glnhisleul euglnleuth rvaltrpgly ilelysglnl euglnthrar gvalleuala ilegluargt yrleulysas pglnglnleu leuglyilet rpglycysse rglylysleu ilecysthrt hralavalpr otrpasnser sertrpsera snglyglygl yglysergly glyglyglys erglualagl nglnhisleu

	leuglnleut hrvaltrpgl yilelysgln leuglnalaa rgvalleual avalgluarg<br>tyrleulysa spglnlysph eleuglyleu trpglycyss erglylysil eilecysthr<br>thralavalp rotrpasnse rthrtrpser asnglyglyg lyglysergl yglyglygly<br>serglualag lnglnhisle uleuglnleu thrvaltrpg lyilelysgl nleuglnala<br>argvalleua lavalgluar gtyrleulys aspglnglnl euleuglyil etrpglycys<br>serglylysh isilecysth rthrasnval protrpasns ersertrpse rasn

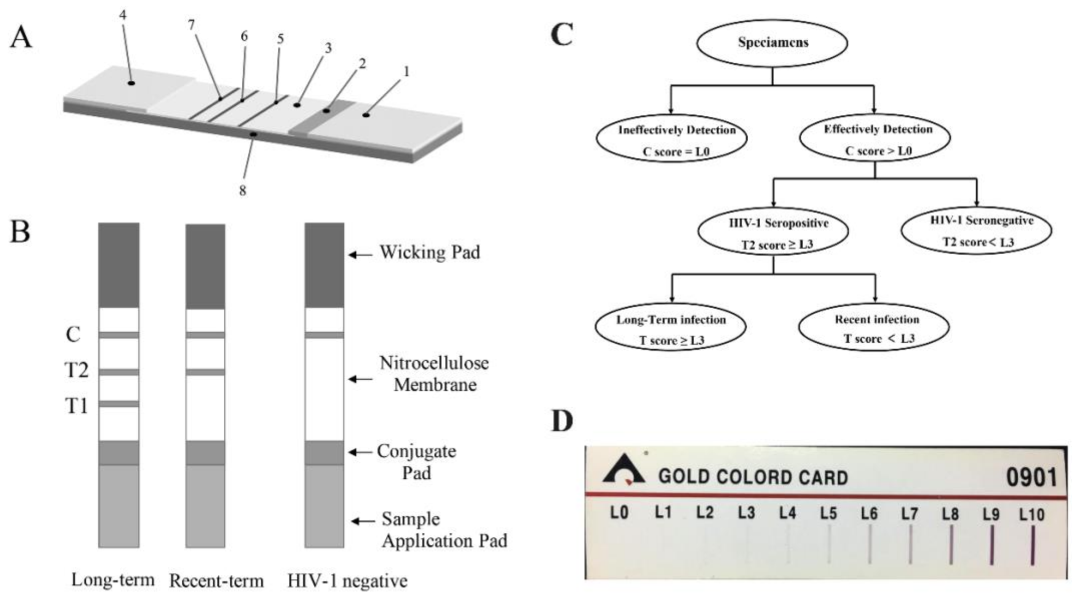

Figure 1. The diagram of the HIV-1 rapid recent-infection testing strip (RRITS). (A) the components of HIV-1 RRITS; 1-8 represent sample application pad, conjugate pad, nitrocellulose membrane, wicking pad, first detection line, second detection line, control line and floor respectively. (B) the results of the HIV-1 RRITS; C, control line; T_2, second testing line; T_1, first testing line. (C) the flow chart used to interpretation of recent and long-term infection. (D) gold colored card (ranged from L0 to L10).
